# The contribution of being physically active to successful aging

**DOI:** 10.3389/fnhum.2023.1274151

**Published:** 2023-11-14

**Authors:** Laura Piccardi, Anna Pecchinenda, Massimiliano Palmiero, Marco Giancola, Maddalena Boccia, Anna Maria Giannini, Cecilia Guariglia

**Affiliations:** ^1^Department of Psychology, Sapienza University of Rome, Rome, Italy; ^2^San Raffaele Cassino Hospital, Cassino, Italy; ^3^Department of Communication Sciences, University of Teramo, Teramo, Italy; ^4^Department of Biotechnological and Applied Clinical Sciences, University of L’Aquila, L’Aquila, Italy; ^5^IRCCS Fondazione Santa Lucia, Rome, Italy

**Keywords:** ageing, prevention, physical activity, working memory, stress, depression, anxiety

## Abstract

Growing old involves changes in physical, psychological, and cognitive functions. Promoting physical and mental health has become one of the priorities for an aging population. Studies have demonstrated the benefits of engaging in regular physical activity. Here, we aimed to understand the relationships between physical activity and working memory complaints in attention, memory storage, and executive functions. We hypothesized that physical activity was negatively associated with complaints in working memory domains after controlling for socio-demographics and distress factors, such as anxiety, stress, and depression. Two hundred and twenty-three individuals aged between 65 and 100 years (74.84; SD = 7.74; 133 males) without self-reported neurological and/or psychiatric disorders completed a questionnaire on socio-demographic, with questions on physical activity and the Italian version of the working memory questionnaire (WMQ) and the DASS-21 measuring anxiety, stress, and depression. Results from three linear regression models showed that low physical activity was associated with complaints in attention (*R*^2^ = 0.35) and executive functions (*R*^2^ = 0.37) but not in memory storage (*R*^2^ = 0.28). Notably, age, gender, and total emotional distress (DASS score) were significant in all regression models. Our results suggested regular physical activity, even just walking, is crucial for maintaining efficient cognitive function. Theoretical and practical implications for engaging in physical activity programs and social aggregation during exercise are considered. Limitations are also presented.

## Introduction

Individuals in industrialized countries live longer, and the number of people aged 80 or older is expected to triple by 2050, reaching 426 million (World Health Organization, 2020). This outlook makes promoting healthy aging a priority, considering the high healthcare costs for old people with pathological aging (Wimo et al., 2013; Persson et al., 2022).

Aging often brings health issues like hearing and vision loss, chronic pain, diabetes, depression, and cognitive decline. As people age, they often experience multiple health conditions simultaneously. These are called geriatric syndromes, complex health states that can arise with older age (see Inouye et al., 2007 for the definition of geriatric syndromes). Several factors are essential for achieving successful aging. For example, the Rowe and Kahn (1997) focuses on factors contributing to healthy aging, including physical and mental health, cognitive functioning, and social engagement. These authors distinguished between usual and successful aging because of diet and physical exercise. Crowther et al. (2002) incorporated into this model positive spirituality that contributes to coping strategies and health promotion. In general, models promoting physical and mental health emphasize the importance of a richly stimulating and active life. It is a virtuous circle in which preserving the older person’s autonomy decreases the risk factors caused by chronic diseases (Nimrod and Ben-Shem, 2015; Liu et al., 2022).

A plethora of studies stressed that engaging in physical activity not only reduces hypertension, diabetes, obesity, insomnia, depression, and anxiety (Ekstrom et al., 2020; Singh et al., 2023) but also triggers cognitive functioning (e.g., Kramer et al., 1999; Colcombe and Kramer, 2003; Winter et al., 2007; Northey et al., 2018; Troisi Lopez et al., 2023). Specifically, exercise significantly enhances memory, attentional processes, and executive-control functions (Lista and Sorrentino, 2010; Fernandes, 2017). Kramer et al. (1999) conducted a study highlighting the significance of physical activity in enhancing executive control and the related brain regions that support this function. Notably, physical exercise positively affects cognitive functioning throughout the life span (for a review, see Mandolesi et al., 2018; Northey et al., 2018; Serra et al., 2021). Indeed, numerous cross-sectional and epidemiological studies have consistently demonstrated the cognitive benefits of physical exercise in both young and older adults (for a review, see Mandolesi et al., 2018; Northey et al., 2018). Specifically, exercise significantly enhances memory, attentional processes, and executive-control functions (Lista and Sorrentino, 2010; Fernandes, 2017). Additionally, a plethora of studies have further solidified the positive impact of engaging in physical activity on various cognitive domains (e.g., Colcombe and Kramer, 2003; Winter et al., 2007; Northey et al., 2018; Troisi Lopez et al., 2023).

Despite the role of physical activity on cognition, age was found to positively affect cognition, particularly attention, memory, executive functions, visuospatial abilities, and environmental and mental representation(e.g., Lezak et al., 2012; Piccardi et al., 2015; Mandolesi et al., 2018; Boccia et al., 2019; Di Vita et al., 2020, 2022; D'Antuono et al., 2022). Importantly, cognitive deficits co-occur in aging with depression (Korczyn and Halperin, 2009; Curran and Loi, 2012; Boccia et al., 2015; Aajami et al., 2020). Specifically, late-life depression (LLD) has been considered a risk factor for dementia (Korczyn and Halperin, 2009; Wu et al., 2020; Ly et al., 2021). Kiloh (1961) coined the term “pseudodementia” to indicate elderly people with depression who were initially misdiagnosed with dementia. Furthermore, individuals suffering from LLD show poor performance in executive functions and episodic memory tasks (Herrmann et al., 2007; Strømnes et al., 2013; Rajtar-Zembaty et al., 2022). As these conditions all hurt cognitive functioning, it is essential to encourage elderly individuals to be physically active daily. Furthermore, physical inactivity represents a risk factor for Alzheimer’s Disease (AD), as exercise increases blood flow to the brain, which promotes neurogenesis and maintains the volume of the hippocampus (Cass, 2017), an essential structure for the consolidation of memories and learning. Sofi et al. (2011), in a meta-analysis including 33,816 individuals without dementia at baseline, found that greater physical activity levels represented a protective factor as they were related to a significant decrease in the onset of dementia. Moreover, in people already suffering from dementia, physical activity helps maintain high functional cognitive levels (Panza et al., 2018). Some researchers also reported that physical activity can prevent the transition from Mild Cognitive Impairment (MCI) to dementia (Nuzum et al., 2020). Physical activity provides beneficial effects in reducing depression, anxiety, and distress symptoms, and it is considered a mainstay approach to managing depression and anxiety (Singh et al., 2023).

Even a routine of daily movements is sufficient for cognitive improvement. Performing physical activity has positive effects also in terms of life satisfaction and acceptance of the aging process (Gellert et al., 2019). Based on 23 longitudinal studies on the relationship between physical activity (i.e., any levels of exercise and activities) and healthy aging, Daskalopoulou et al. (2017) found an increase of almost 40% in self-reported healthy aging compared to older people who do not perform physical activities. Moreno-Agostino et al. (2020) found that physically inactive older individuals quickly exhibit worse trajectories of age-related health no matter the degree of physical activity. Physical exercise is associated with a global improvement in cognition (Mandolesi et al., 2018). In their recent narrative review, Szychowska and Drygas (2022) emphasize the crucial role of physical activity in maintaining physical and cognitive function and promoting mental well-being. They highlight the positive impact of physical activity on social engagement, which is linked to healthy aging.

In contrast, physical activity does not appear to improve specific cognitive sub-domains such as memory, executive functions, and attention (Song et al., 2018). More specifically, when assessing the effects of different modalities of physical exercise, Song et al. (2018) highlight the importance of aerobic exercise in improving cognitive health, as it improves cardiovascular functioning, which leads to an increase in cerebral blood flow and consequently leads to an increase of oxygen and glucose to the brain tissue, enhancing neurotransmitter availability and neural efficiency (Ainslie et al., 2008; Chaudhary et al., 2010). Furthermore, cardiovascular fitness is also related to reducing vascular risk factors associated with cognitive decline (Aspenes et al., 2011; Donley et al., 2014). Finally, general physical activity may also produce several positive effects from a social and psychological point of view, mainly when individuals perform such activity together. In this case, engaging in physical activities in the company of others may also help establish social connections and counteract loneliness. Indeed, Pecchinenda et al. (2023) found that older individuals who had several cultural and sports memberships and were more often engaging in physical activities (e.g., exercising, walking, dancing) reported less loneliness and emotional problems.

Based on these premises, we assessed the relationship between physical activity (including different forms of physical activity from daily walking and dancing to more intense activities such as trekking) and complaints in the attention, memory storage, and executive functions of working memory. Following the working memory multicomponent model (Baddeley and Hitch, 1974; Baddeley, 2000), working memory into three components: the central executive, the phonological loop, and the visuo-spatial sketchpad. The first component helps to focus attention on important information and coordinates cognitive processes when multitasking. It also oversees the other two components. The phonological loop stores phonological information and prevents decaying by frequently refreshing information in a rehearsal loop—the visuospatial sketchpad stores visual and spatial information.

Additionally, a fourth component called the episodic buffer has been included (Baddeley, 2000). It holds representations that combine phonological, visual, and spatial information, forming a unified episodic representation. Sometimes people forget things quickly after seeing or hearing them, like a recent recall or what they were looking for when they enter a room. If someone cannot remember things, it could be an early sign of AD. As people get older, their memory may not be as good at remembering things for a short time or holding a lot of information (Baddeley et al., 1991; Logie et al., 2004).

There are only a few tools available to evaluate the effectiveness of working memory in everyday activities. One of these tools is the Working Memory Questionnaire (WMQ), created and validated by Vallat-Azouvi et al. (2012). The WMQ has also been translated and validated in Italian by Guariglia et al. (2020). The WMQ is a reliable tool that can distinguish between patients with brain injury and healthy individuals. It also has an ecological validity, meaning that an individual’s performance on the test reflects their real-world abilities. Furthermore, Guariglia et al. (2020) showed a strong correlation between self-assessment and performance on a paper and pencil test.

Understanding the connection between physical activity and different parts of working memory is important for preventing atypical aging. Our main objective in this paper is to further investigate the association between physical activity and working memory. Specifically, to investigate the effects of general physical activity on a validated questionnaire of day-to-day functional working memory problems in three distinct domains (attention, storage, and executive functions). We have paid particular attention to visuospatial working memory due to its significance in spatial navigation and route learning, which are vital for independence in day-to-day activities. It also plays a crucial role in executive functions and daily decision-making, including decisions that could impact economic choices. Moreover, even though working memory declines as age increases, it plays a critical role in explaining the efficacy of cognitive training in older adults (e.g., Costello and Buss, 2018; Matysiak et al., 2019).

We developed the hypothesis that physical activity can be associated negatively with complaints in working memory domains such as attention, storage, and executive control, after controlling for socio-demographics, anxiety, stress, and depression.

## Method

### Participants

The eligible study sample comprised 223 individuals without neurological or psychiatric disorders (age range 65–100 years; mean age = 74.84, SD = 7.41; 133 males, 90 females). The sample was partially overlapped with that described by Pecchinenda et al. (2023). Participants had an education level ranging from 0 to 22 years (mean = 7.4, SD = 3.73). They were members of Age Italia associations (i.e., a group of associations working in the field of social policies for well-being in old age), and they voluntarily took part in the study. Exclusion criteria were any neurological^1^ or major psychiatric illness, the use of psychotropic medications, previous traumatic brain injury, history of learning disabilities, and alcohol or drug abuse. The absence of these conditions was verified through the answers to a health-socio-demographic questionnaire filled in at the beginning of the study. The study was approved by the Local Ethical Committee following the Declaration of Helsinki. Each participant provided informed consent before taking part in the study.

### Instruments

We used a health-socio-demographic questionnaire developed by the AGE Italia Association for their members. Physical activity was evaluated by one item along a 3-point Likert scale ranging from 0 (never) to 2 (often), in which participants could also indicate what kind of physical activity they carried out and how often they did it. Furthermore, participants completed the Italian version of the Working Memory Questionnaire (WMQ: Guariglia et al., 2020), aimed to assess self-perceived working memory deficits in everyday life. The test consists of 30 questions that assess three domains of working memory: attention, memory storage, and executive functions. Each question is scored on a five-point Likert scale, ranging from 0 (“no problem at all”) to 4 (“very severe problem in everyday life”). Three sub-scores (maximal score 40 for each subscale) and a total score (out of 120) are computed. Higher scores correspond to more complaints. The memory storage domain corresponds to the ability to maintain information in short-term memory for a short time but also the ability to perform mental calculations and written text comprehension. The attention domain assesses distractibility, mental slowness, mental fatigue, and dual-task processing. The third domain is related to executive aspects of working memory, such as decision-making, planning, or shifting. Participants were also asked to complete the Italian version of the Depression Anxiety Stress Scale (DASS-21, Bottesi et al., 2015). It is composed of a 21-item questionnaire based on a four-point rating scale (i.e., ranging from 0 = “did not apply to me at all” to 3 = “applied to me very much, or most of the time), and it is aimed at assessing three constructs: anxiety, depression, and stress. The higher the score, the more severe the emotional distress was.

### Procedure

All instruments were distributed through the AGE Italia associations (for the complete list, see Acknowledgments) in May–June 2021. Participants first completed the health-socio-demographic questionnaire, and if they agreed to participate in this study, they were asked to complete the DASS and WMQ scales after providing informed consent. Each association collected the completed paper questionnaires, converted them to PDF files, and sent them to the researchers. Data collection was completed by the end of September 2021.

### Data analysis

Analyses were performed using IBM SPSS Statistics software v.24 (2016). First, z-scores were computed to check for potential univariate outliers. Data were checked also for normality, and then, a preliminary correlation analysis was performed. Finally, three regression analyses were conducted, considering physical activity as the independent variable and attention, storage, and executive functions as the outcomes. Three regression models were advanced, entering the outcomes one by one.

## Results

A cut-off of ±4 z-scores was used as the reference values as the initial sample was larger than 100 participants (see Mertler and Vannatta, 2005; Giancola et al., 2023). No outlier was detected. The Kolmogorov–Smirnov Test was performed to check for normal distribution, showing that all variables were not normally distributed: Z_Age_ = 0.138, *p* < 0.0001; Z_Physical activity_ = 0.00, *p* < 0.05, Z_Attention_ = 0.105, *p* < 0.0001; Z_Memory Storage_ = 0.080, *p* < 0.005; Z_Executive Functions_ = 0.080, *p* < 0.05; Z_Stress_ = 0.124, *p* < 0.0001; Z_Anxiety_ = 0.163, *p* < 0.0001; Z_Depression_ = 0.158, *p* < 0.0001; Z_Total DASS_ = 0.124, *p* < 0.0001. Then, the Spearman Rho correlational analysis (see Table 1) shows that physical activity correlates negatively with complaints in attention (*r* = −0.183; *p* < 0.01), memory storage (*r* = −0.179; *p* < 0.01), and executive functions (*r* = −0.278; *p* < 0.01). For the covariates, age correlates positively with complaints in attention (*r* = 0.308; *p* < 0.01), memory storage (*r* = 0.257; *p* < 0.01), and executive functions (*r* = 0.207; *p* < 0.01). Gender (females = 1, males = 0) correlates positively with complaints in attention (*r* = 0.207; *p* < 0.01), memory storage (*r* = 0.198; *p* < 0.01) and executive functions (*r* = 0.243; *p* < 0.01). Also, educational level correlates negatively with complaints in attention (*r* = −0.168; *p* < 0.01), memory storage (*r* = −0.194; *p* < 0.01), and executive functions (*r* = −0.187; *p* < 0.01). Finally, the total DASS correlates positively with complaints in attention (*r* = 0.486; *p* < 0.01), memory storage (*r* = 0.480; *p* < 0.01), and executive functions (*r* = 0.558; *p* < 0.01).

**Table 1:** Means, standard deviations, and correlations among study variables.

	Mean (SD)	1. Age	2. Gen	3. Edu.	4. P. Act.	5. Att.	6. Mem. Sto.	7. Exe.	8. Str.	9. Anx.	10. Dep.	11. T. DASS
1.	74.84 (7.4)	1										
2.	–	0.077	1									
3.	7.4 (3.73)	−0.405**	−0.175^**^	1								
4.	0.80 (0.78)	−0.075	−0.106	0.198^**^	1							
5.	11.4 (6.25)	0.308**	0.207^**^	−0.168^*^	−0.183^**^	1						
6.	10.7 (6.85)	0.257**	0.198^**^	−0.194^**^	−0.179^**^	0.859^**^	1					
7.	11.39 (6.03)	0.207**	0.243^**^	−0.187^**^	−0.278^**^	0.717^**^	0.743^**^	1				
8.	4.80 (3.08)	0.037	0.027	−0.106	−0.003	0.401^**^	0.376^**^	0.416^**^	1			
9.	3.08 (2.87)	0.164*	0.137^*^	−0.153^*^	−0.168^*^	0.432^**^	0.451^**^	0.573^**^	0.609^**^	1		
10.	2.86 (2.85)	0.107	0.078	−0.101	−0.020	0.446^**^	0.445^**^	0.442^**^	0.676^**^	0.542^**^	1	
11.	10.74 (7.67)	0.109	0.106	−0.142^*^	−0.092	0.486^**^	0.480^**^	0.558^**^	0.881^**^	0.542^**^	0.843**	1

^**^ *p* < 0.01 (two-tailed); ^*^ *p* < 0.05 (two-tailed); Gen, Gender; Edu, Educational Level; P. Att, Physical Activity; Att, Attention; Mem, Memory; Sto, Storage; Exe, Executive Functions; Str, Stress; Anx, Anxiety; Dep, Depression; DASS, Total Depression Anxiety Stress Scale.

**Figure 1:**
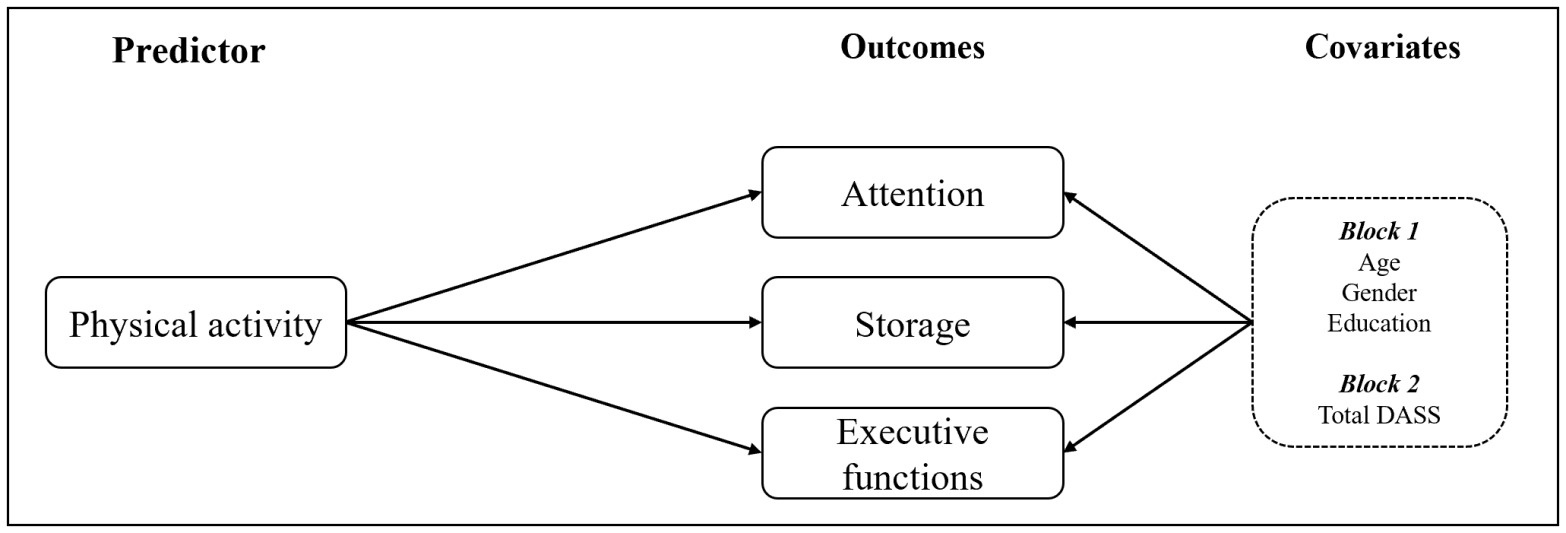
The theoretical model advanced in the current study.

To test the hypothesis that physical activity is associated with complaints in working memory domains (i.e., attention, storage, and executive functions), three linear hierarchical regressions, one for each component of working memory, were tested. Socio-demographics (age, gender, and educational level) and emotional distress, as indexed by the total DASS score (stress, anxiety, and depression), were included as covariates (see Figure 1). Although data were not normally distributed, to test the causal relationship between physical activity and complaints in working memory, a linear regression model was applied as the violation of the normality assumption in linear regression analyses does not bias point estimates or tests in large sample sizes (see Schmidt and Finan, 2018). Then, to check for possible multicollinearity among independent variables (age, gender, education level, total DASS, and physical activity), the variance inflation factor (VIF) was checked: values ranged across variables from 1.035 to 1.217, considering the three regression models. Given the suggested cut-off value of 5 (see Sheather, 2009; Hair et al., 2010), no multicollinearity was found.

**Figure 2:**
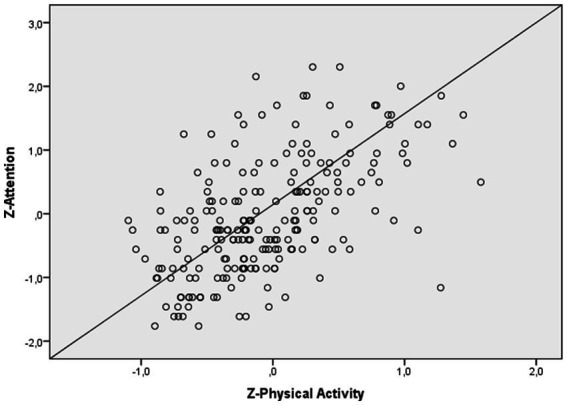
Relationship between physical activity and attention of the working memory.

Regarding complaints in attention (see Figure 2), the first model was significant, and explained 13.2% of the variance (*R^2^* = 0.132; *R^2^* adjusted = 0.120, *p* < 0.0001). After introducing the total DASS score, the second model was significant and explained 33.2% of the variance (*R^2^* = 0.332; *R^2^* adjusted = 0.320, *p* < 0.0001), which is an additional 20% of the variance (*R^2^* change = 0.320; *p* < 0.0001). After introducing the physical activity score, the third model was significant, and explained 34.7% of the variance (*R^2^* = 0.347; *R^2^* adjusted = 0.332, *p* < 0.0001), which is an additional 1.5% of the variance (*R^2^* change = 0.332; *p* < 0.5). Physical activity was significant (*β* = −0.125, *p* < 0.05). Finally, age (*β* = 0.258, *p < 0.*0001), gender (*β* = 0.112, *p* < 0.05) and total DASS (*β* = 0.452, *p* < 0.001) were also significant.

**Figure 3:**
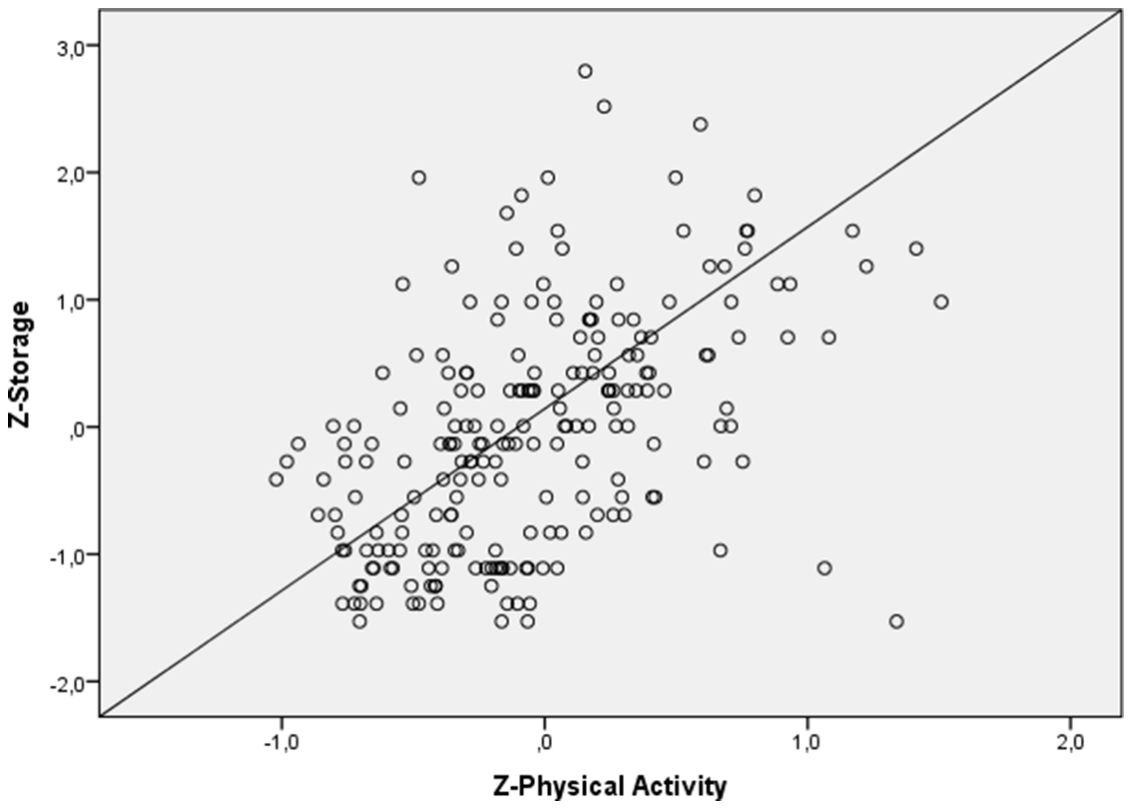
Relationship between physical activity and storage of working memory.

Regarding complaints in storage (see Figure 3), the first model was significant and explained 10.9% of the variance (*R^2^* = 0.109; *R^2^* adjusted = 0.097, *p* < 0.0001). After adding the total DASS score, the second model was significant and explained 27.3% of the variance (*R^2^* = 0.273; *R^2^* adjusted = 0.26, *p* < 0.0001), which is an additional 16.4% of the variance (*R^2^* change = 0.26; *p* < 0.0001). After adding the physical activity score, the third model was significant and explained 28.1% of the variance (*R^2^* = 0.281; *R^2^* adjusted = 0.264, *p* < 0.0001), which is an additional 1.2% of the variance (*R^2^* change = 0.264; *p <* 0.05). Physical activity was not significant (*β* = −0.091, *p <* 0.05). Finally, age (*β* = 0.172, *t* = 2.782, *p < 0.*001), gender (*β* = 0.122, *p* < 0.05) and total DASS (*β* = 0.409, *p* < 0.0001) were significant.

**Figure 4:**
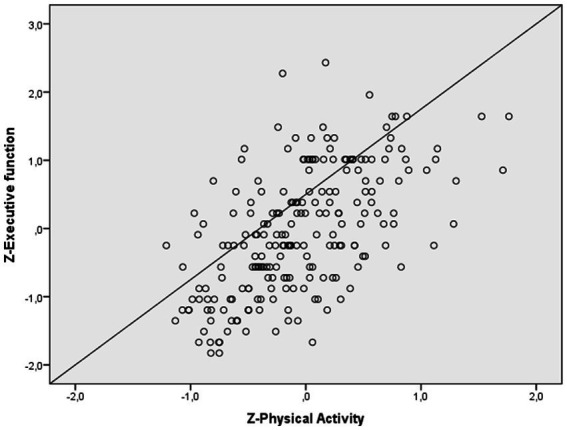
Relationship between physical activity and executive function of working memory.

Regarding complaints in executive function (see Figure 4), the first model was significant, and explained 11.5% of the variance (*R^2^* = 0.115; *R^2^* adjusted = 0.102, *p* < 0.0001). After adding the total DASS score, the second model was significant and explained 33.8% of the variance (*R^2^* = 0.338; *R^2^* adjusted = 0.325, *p* < 0.0001), which is an additional 22.3% of the variance (*R^2^* change = 1.218, *p* < 0.0001). After adding the physical activity score, the third model was significant and explained 37.4% of the variance (*R^2^* = 0.374; *R^2^* adjusted = 0.360, *p* < 0.0001), which is an additional 3.6% of the variance (*R^2^* change = 0.36; *p < 0*.0001). Physical activity was significant (*β* = −0.196, *p < 0*.0001). Finally, age (*β* = 0.141, *p < 0.*05), gender (*β* = 0.158, *t* = 2.880, *p* < 0.01) and total DASS (*β* = 0.475, *p* < 0.0001) were also significant.

## Discussion

Our goal was to conduct an investigation into the associations between physical activity and working memory complaints in attention, memory storage, and executive functions in a sample of older people. We hypothesized that older individuals who engage in higher levels of physical activity would experience fewer complaints related to working memory, regardless of their socio-demographic background, as well as the presence of anxiety, stress, and depression.

Our findings confirmed that physical activity was associated with fewer complaints in attention (*R*^2^ = 0.35) and executive functions (*R*^2^ = 0.37) but not in memory storage (*R*^2^ = 0.28). Based on the effect sizes (R^2^) our results support the idea that physical activity represents a critical factor in explaining some working memory complaints. Notably, despite the minimal difference, the higher *R*^2^ for executive functions could be explained by considering the physical activity levels. Indeed, whereas attention mainly benefits from acute physical activity, executive functions, involving decision-making, planning, and shifting, are more susceptible to improvement through longitudinal exercise (De Greeff et al., 2018), as probably occurred in the present sample. However, such an interpretation should be confirmed by further studies. These results confirmed previous research, and highlight that engaging in regular physical activity yields a favorable effect on attention and executive functions of working memory, particularly in the anterior regions of the brain. In this vein, a Hewston et al. (2021) conducted a systematic review and meta-analysis to examine the impact of dance on the cognitive abilities of older adults across seven domains (global cognitive function, executive function, learning and memory, complex attention, language, social cognition, and perceptual-motor function). The study suggested that dance is likely to enhance global cognitive function, but it does not seem to affect complex attention. It may have little to no effect on executive function, although there could be an improvement in older adults with mild cognitive function. In this study, it is unclear which activity specifically resulted in the reported improvements in attention and executive functions of working memory. With respect to dance, it involves actively keeping track of steps and focusing on one’s partner or instructor. At the same time, walking is a more passive activity that can benefit the cardiovascular system and consecutively benefits effects on brain activity. It is worth noting that our findings indicate that consistent exercise impacts executive control processes that are linked to the prefrontal and frontal brain regions. These areas of the brain experience significant and disproportionate changes as age increases (see Kramer et al., 1999). While we cannot categorize the physical activity of our participants, we can confirm that they all engaged in aerobic exercises. Research by Kramer et al. in 1999 has shown that these activities are more effective than anaerobic exercises such as stretching and muscle toning.

In general, the present findings suggest that being active is essential regardless of the type of motor or intensity of activity. However, some forms of motor activity benefit cognitive functions more than others because they involve recreational and social conditions. For example, older people who report dancing tend to feel less lonely and less depressed (Pecchinenda et al., 2023). On the other hand, a growing body of literature shows that physical activity represents a vital lifestyle factor for healthy aging (e.g., Dias et al., 2017; Amer and Rasheedy, 2018; Moreno-Agostino et al., 2020). Physical activity produces beneficial effects by reducing coronary heart disease and hypertension and improving glycemic control. It also enhances balance, which helps prevent falls and fractures that lead to the elderly being bedridden for long periods and a consequent decline in all cognitive processes, reducing autonomy and the beneficial effects of everyday life lost due to forced immobility. Some other studies demonstrate a crucial role in protecting against cognitive decline and neurological disorders (e.g., Abbott et al., 2004; Weuve et al., 2004; McDermott et al., 2006; Amer and Rasheedy, 2018; Iso-Markku et al., 2022; Kantawala et al., 2023).

According to Kaeberlein (2018), physical activity extends the life span (the time an individual lives) and the health span (the healthy time an individual lives). Interestingly, muscle strengthening and aerobic activity reduce the risk of developing Alzheimer’s Disease (AD) and improve performance on cognitive tests (Boyle et al., 2009; Buchman et al., 2012; Ekstrom et al., 2020). However, several studies also show that walking is enough to promote cognitive and psychological health and that walking has several advantages, including that it is easily accessible. Indeed, in our sample, walking was one of the most reported physical activities as it may involve an aerobic activity that is low-cost and accessible; it can be performed alone or in groups, outdoors or indoors (Reitlo et al., 2018). Probably, this is also due to the great popularity of pedometers, step counters, and fitness watches that provide information on the wearer’s health, including counting kilometers traveled and steps taken, which for many represent a positive reinforcement for the physical activity performed. In our sample, women reported being more active than men and self-reported better cognitive health. Physical activity also helped in reducing perceived distress. Our data support the importance of staying physically active and how this behavior has positive effects on perceived attention and executive functions as well as on mental health. In general, practicing physical activity reduces anxiety and depression levels and improves cognitive health.

The present study took place during the COVID-19 pandemic, which led to a reduction in certain lifestyle habits. Undoubtedly, older individuals who practiced physical activity by attending gym classes had to stop, as did those who danced; all physical activities that required amateur performance were also stopped. Most likely, the adverse effects of these interruptions are less visible in the immediate but will be visible in the near future. However, the pandemic has also seen the flourish of alternatives, such as exercise classes being delivered via the Internet, even in our homes with customized programs. This digital revolution may have the advantage of making physical activity part of daily routines, albeit it carries the risk of reducing the opportunities for meeting and socializing that going to the gym may provide. Home-fitness activity has also been adopted by many people to counter negative feelings such as fear and sadness resulting from isolation and news concerning the virus. According to Capriotti et al. (2022), engaging in physical activity at home can be a great way for older adults to prevent age-related issues. This exercise has numerous benefits, including reducing the risk of falls, preventing loss of independence and frailty, and improving physical abilities such as balance, strength, mobility, flexibility, and aerobic capacity. It can also positively affect cognitive functions while boosting overall mood and well-being. Although the data we collected covered a critical time with respect to COVID-19, we cannot quantify the effects of lifestyle adjustments or particular physical activities. Our findings just suggest that those who participate in physical activity generally encounter fewer difficulties with attention and executive functions, regardless of the pandemic conditions.

To conclude with Juvenal’s words, the present findings indicate that *‘Mens sana in corpore sano*’ (a sound mind in a sound body), suggesting the importance of any physical activity on perceived cognitive and emotional health, particularly in maintaining good perceived attention and executive functions of working memory.

## Limitations and conclusions

We would like to acknowledge some limitations of the present study. First, even though we collected data concerning cognitive functioning by a reliable instrument such as the WMQ, the self-reporting of cognitive performance could be affected by psychological mental health, such as late-life depression or anxiety. Therefore, future studies should use both subjective and objective assessments of the cognitive and emotional profile of older individuals as well as subjective and objective measures of physical activity (e.g., type, frequency, duration, psychophysical parameters, and so forth). Although the study has value, it is important to acknowledge its limitations. Second, we evaluate cognitive functioning only by considering complaints in working memory. To get a more granular evaluation of cognition in older people, further studies should consider the key role of physical activity in other cognitive functions, such as flexibility and inhibition, planning, reasoning, and problem-solving abilities, requiring finding multiple, appropriate, and novel solutions (Giancola et al., 2022a,b). In this direction, divergent thinking is slightly preserved in aging (for example, Palmiero, 2015; Palmiero et al., 2017), and, therefore, it could be interesting to explore whether it can be coupled with physical activity and influence cognitive and emotional well-being.

Finally, the impact of the COVID-19 pandemic on participants’ cognitive functioning could not be fully explored. We did not inquire about changes in participants’ habits before and after COVID-19. However, we can speculate that the pandemic may have influenced decisions to engage in light physical activity outdoors rather than indoors and to reduce contact with others as recommended for vulnerable populations. While this recommendation promoted safe behavior, it could also lead to loneliness, which has negative effects on overall health.

## Data availability statement

The raw data supporting the conclusions of this article will be made available by the authors, without undue reservation.

## Ethics statement

The studies involving humans were approved by Department of Psychology, Sapienza University of Rome, Italy. The studies were conducted in accordance with the local legislation and institutional requirements. The participants provided their written informed consent to participate in this study.

## Author contributions

LP: Conceptualization, Data curation, Methodology, Project administration, Supervision, Writing – original draft, Writing – review & editing. AP: Conceptualization, Data curation, Funding acquisition, Project administration, Writing – review & editing. MP: Data curation, Formal analysis, Writing – review & editing. MG: Data curation, Formal analysis, Writing – review & editing. MB: Conceptualization, Methodology, Writing – review & editing. AG: Conceptualization, Project administration, Supervision, Writing – review & editing. CG: Conceptualization, Funding acquisition, Project administration, Supervision, Writing – review & editing.

## Footnotes

^1^We considered as ‘exclusion criteria’ those proposed by Crook et al. (1986)—i.e., a history of head trauma, a history of brain fever, dementia (more specifically, individuals who had been diagnosed with mild cognitive impairment, Alzheimer’s disease (AD), or any other form of dementia by a healthcare provider), or any other state of altered consciousness, use of benzodiazepines in the previous 3 months, use of illicit drugs, any visual, auditory or motor impairments, any symptomatic cardiovascular conditions. Moreover, those who had an unstable medical condition (such as hospitalization within the last 6 weeks or frequent emergency room visits).

## References

[ref1] Aajami Z. Kazazi L. Toroski M. Bahrami M. Borhaninejad V. (2020). Relationship between depression and cognitive impairment among elderly: a cross-sectional study. J. Caring Sci. 9, 148–153. doi: 10.34172/jcs.2020.022, PMID: ; PMCID: PMC7492969 32963983 PMC7492969

[ref2] Abbott R. D. White L. R. Ross G. W. Masaki K. H. Curb J. D. Petrovitch H. (2004). Walking and dementia in physically capable elderly men. JAMA 292, 1447–1453. doi: 10.1001/jama.292.12.1447 15383515

[ref3] Ainslie P. N. Cotter J. D. George K. P. Lucas S. Murrell C. Shave R.. (2008). Elevation in cerebral blood flow velocity with aerobic fitness throughout healthy human ageing. J. Physiol. 586, 4005–4010. doi: 10.1113/jphysiol.2008.158279 18635643 PMC2538930

[ref5] Amer M. S. Rasheedy D. (2018). Physical activity: evidence for the anti-ageing effects in elderly. Med. Bull. 23, 29–32.

[ref6] Aspenes S. Nilsen T. Skaug E. Bertheussen G. Ellingsen O. Vatten L.. (2011). Peak oxygen uptake and cardiovascular risk factors in 4631 healthy women and men. Med. Sci. Sports Exerc. 43, 1465–1473. doi: 10.1249/MSS.0b013e31820ca81c 21228724

[ref7] Baddeley A. (2000). The episodic buffer: a new component of working memory? Trends Cogn. Sci. 4, 417–423. doi: 10.1016/S1364-6613(00)01538-2 11058819

[ref8] Baddeley A. D. Bres S. Della Sala S. Logie R. Spinnler H. (1991). The decline of working memory in Alzheimer’s disease. A longitudinal study. Brain 114, 2521–2542. doi: 10.1093/brain/114.6.2521 1782529

[ref9] Baddeley A. Hitch G. (1974). “The psychology of learning and motivation: advances in research and theory” in Working memory: the psychology of learning and motivation. ed. Bower G. H. (New York: Academic Press), 47–89.

[ref10] Boccia M. Acierno M. Piccardi L. (2015). Neuroanatomy of Alzheimer’s disease and late-life depression: a coordinate-based meta-analysis of MRI studies. J. Alzheimers Dis. 46, 963–970. doi: 10.3233/JAD-142955 25869784

[ref11] Boccia M. Di Vita A. Diana S. Margiotta R. Imbriano L. Rendace L.. (2019). Is losing one’s way a sign of cognitive decay? Topographical memory deficit as an early marker of pathological aging. J. Alzheimers Dis. 68, 679–693. doi: 10.3233/JAD-180890 30883347

[ref12] Bottesi G. Ghisi M. Altoè G. Conforti E. Melli G. Sica C. (2015). The Italian version of the depression anxiety stress Scales-21: factor structure and psychometric properties on community and clinical samples. Compr. Psychiatry 60, 170–181. doi: 10.1016/j.comppsych.2015.04.005 25933937

[ref13] Boyle P. A. Buchman A. S. Wilson R. S. Leurgans S. E. Bennett D. A. (2009). Association of muscle strength with the risk of Alzheimer disease and the rate of cognitive decline in community-dwelling older persons. Arch. Neurol. 66, 1339–1344. doi: 10.1001/archneurol.2009.240 19901164 PMC2838435

[ref14] Buchman A. S. Boyle P. A. Yu L. Shah R. C. Wilson R. S. Bennett D. A. (2012). Total daily physical activity and the risk of AD and cognitive decline in older adults. Neurology 78, 1323–1329. doi: 10.1212/WNL.0b013e3182535d35 22517108 PMC3335448

[ref15] Capriotti A. Patregnani V. Federici A. (2022). Home-fitness and active ageing: A review. Sci. J. Sport Perform. 1, 167–178. doi: 10.55860/IIUS3216

[ref16] Cass S. P. (2017). Alzheimer’s disease and exercise: a literature review. Curr. Sports Med. Rep. 16, 19–22. doi: 10.1249/JSR.0000000000000332 28067736

[ref17] Chaudhary S. Kang M. K. Sandhu J. S. (2010). The effects of aerobic versus resistance training on cardiovascular fitness in obese sedentary females. Asian J. Sports Med. 1, 177–184. doi: 10.5812/asjsm.34835 22375205 PMC3289184

[ref18] Colcombe S. Kramer A. F. (2003). Fitness effects on the cognitive function of older adults. Psychol. Sci. 14, 125–130. doi: 10.1111/1467-9280.t01-1-01430 12661673

[ref19] Costello M. C. Buss A. T. (2018, 2018). Age-related decline of visual working memory: Behavioral results simulated with a dynamic neural field model. J. Cogn. Neurosci. 30, 1532–1548. doi: 10.1162/jocn_a_01293 29877766

[ref20] Crook T. Bartus R. T. Ferris S. H. Whitehouse P. Cohen G. D. Gershon S. (1986). Age-associated memory impairment: proposed diagnos- tic criteria and measures of clinical change. Report of a National Institute of Mental Health work group. Dev. Neuropsychol. 2, 261–276.

[ref21] Crowther M. R. Parker M. W. Achenbaum W. A. Larimore W. L. Koenig H. G. (2002). Rowe and Kahn’s model of successful aging revisited: positive spirituality—the forgotten factor. The Gerontologist 42, 613–620. doi: 10.1093/geront/42.5.613 12351796

[ref22] Curran E. M. Loi S. (2012). Depression and dementia. MJA Open 1 Suppl 4, 40–43.

[ref23] D'Antuono G. Maini M. Marin D. Boccia M. Piccardi L. (2022). Effect of ageing on verbal and visuo-spatial working memory: evidence from 880 individuals. Appl. Neuropsychol. Adult 29, 193–202. doi: 10.1080/23279095.2020.1732979 32125884

[ref24] Daskalopoulou C. Stubbs B. Kralj C. Koukounari A. Prince M. Prina A. M. (2017). Physical activity and healthy ageing: a systematic review and meta-analysis of longitudinal cohort studies. Ageing Res. Rev. 38, 6–17. doi: 10.1016/j.arr.2017.06.003 28648951

[ref25] De Greeff J. W. Bosker R. J. Oosterlaan J. Visscher C. Hartman E. (2018). Effects of physical activity on executive functions, attention and academic performance in preadolescent children: a meta-analysis. J. Sci. Med. Sport 21, 501–507. doi: 10.1016/j.jsams.2017.09.595 29054748

[ref26] Di Vita A. D’Antonio F. Boccia M. Lisi S. Di Savino C. Piccardi L.. (2020). Visual mental imagery in mild cognitive impairment: a pilot study. Alzheimers Dement. 16:e045103. doi: 10.1002/alz.045103

[ref27] Di Vita A. Vecchione F. Boccia M. Bocchi A. Cinelli M. C. Mirino P.. (2022). DiaNe: a new first level computerized tool assessing memory, attention, and visuo-spatial processing to detect early pathological cognitive decline. J. Alzheimers Dis. 86, 891–904. doi: 10.3233/JAD-215294 35147537

[ref28] Dias G. Couceiro M. Mendes P. de Lurdes Almeida M. (2017). “Physical activity benefits in active ageing” in Active ageing and physical activity. Springer briefs in well-being and quality of life research (Cham: Springer). doi: 10.1007/978-3-319-52063-6_2

[ref29] Donley D. Fournier S. B. Reger B. Devallance E. Bonner D. Olfert I.. (2014). Aerobic exercise training reduces arterial stiffness in metabolic syndrome. J. Appl. Physiol. 116, 1396–1404. doi: 10.1152/japplphysiol.00151.2014 24744384 PMC4044399

[ref30] Ekstrom E. Neukam S. Kalin L. Wright J. (2020). Physical activity and healthy aging. Clin. Geriatr. Med. 36, 671–683. doi: 10.1016/j.cger.2020.06.009 33010902

[ref002] Fernandes J. Arida R. M. Gomez-Pinilla F. (2017). Physical exercise as an epigenetic modulator of brain plasticity and cognition. Neurosci. Biobehav. Rev. 80, 443–456. doi: 10.1016/j.neubiorev.2017.06.012 28666827 PMC5705447

[ref31] Gellert P. Wienert J. Ziegelmann J. P. Kuhlmey A. (2019). Profiles of physical activity biographies in relation to life and aging satisfaction in older adults: longitudinal findings. Eur. Rev. Aging Phys. Act. 16:14. doi: 10.1186/s11556-019-0221-6 31417662 PMC6689165

[ref32] Giancola M. Palmiero M. Bocchi A. Piccardi L. Nori R. D’Amico S. (2022b). Divergent thinking in Italian elementary school children: the key role of probabilistic reasoning style. Cogn. Process. 23, 637–645. doi: 10.1007/s10339-022-01104-2 35881317

[ref33] Giancola M. Palmiero M. D’Amico S. (2023). Dark triad and COVID-19 vaccine hesitancy: the role of conspiracy beliefs and risk perception. Curr. Psychol., 31, 1–13. doi: 10.1007/s12144-023-04609-x PMC10064627 37359671

[ref34] Giancola M. Palmiero M. Piccardi L. D’Amico S. (2022a). The relationships between cognitive styles and creativity: the role of field dependence-independence on visual creative production. Behav. Sci. 12:212. doi: 10.3390/bs12070212 35877282 PMC9312302

[ref35] Guariglia P. Giaimo F. Palmiero M. Piccardi L. (2020). Normative data and validation of the Italian translation of the working memory questionnaire (WMQ). Appl. Neuropsychol. Adult 27, 376–389. doi: 10.1080/23279095.2018.1552147 30760034

[ref36] Hair J. F. Black W. C. Babin B. J. Anderson R. E. Tatham R. L. (2010). Multivariate data analysis, 7th; Pearson: New York, NY, USA.

[ref38] Herrmann L. L. Goodwin G. M. Ebmeier K. P. (2007). The cognitive neuropsychology of depression in the elderly. Psychol. Med. 37, 1693–1702. doi: 10.1017/S0033291707001134 17610767

[ref39] Hewston P. Kennedy C. C. Borhan S. Merom D. Santaguida P. Ioannidis G.. (2021). Effects of dance on cognitive function in older adults: a systematic review and meta-analysis. Age Ageing 50, 1084–1092. doi: 10.1093/ageing/afaa270, PMID: 33338209

[ref41] Inouye S. K. Studenski S. Tinetti M. E. Kuchel G. A. (2007). Geriatric syndromes: clinical, research, and policy implications of a core geriatric concept. J. Am. Geriatr. Soc. 55, 780–791. doi: 10.1111/j.1532-5415.2007.01156.x 17493201 PMC2409147

[ref42] Iso-Markku P. Kujala U. M. Knittle K. Polet J. Vuoksimaa E. Waller K. (2022). Physical activity as a protective factor for dementia and Alzheimer's disease: systematic review, meta-analysis and quality assessment of cohort and case-control studies. Br. J. Sports Med. 56, 701–709. doi: 10.1136/bjsports-2021-104981 35301183 PMC9163715

[ref43] Kaeberlein M. (2018). How healthy is the healthspan concept? Geroscience 40, 361–364. doi: 10.1007/s11357-018-0036-9 30084059 PMC6136295

[ref44] Kantawala B. Ramadan N. Hassan Y. Fawaz V. Mugisha N. Nazir A.. (2023). Physical activity intervention for the prevention of neurological diseases. Health Sci. Rep. 6:e1524. doi: 10.1002/hsr2.1524 37614284 PMC10442603

[ref45] Kiloh L. G. (1961). Pseudo-dementia. Acta Psychiatr. Scand. 37, 336–351. doi: 10.1111/j.1600-0447.1961.tb07367.x 14455934

[ref46] Korczyn A. D. Halperin I. (2009). Depression and dementia. J. Neurol. Sci. 283, 139–142. doi: 10.1016/j.jns.2009.02.346 19345960

[ref47] Kramer A. F. Hahn S. Cohen N. J. Banich M. T. McAuley E. Harrison C. R.. (1999). Ageing, fitness and neurocognitive function. Nature 400, 418–419. doi: 10.1038/22682 10440369

[ref48] Lezak M. D. Howieson D. B. Bigler E. D. Tranel D. (2012). Neuropsychological assessment. 5th New York, NY: Oxford University Press.

[ref001] Lista I. Sorrentino G. (2010). Biological mechanisms of physical activity in preventing cognitive decline. Cell. Mol. Neurobiol. 30, 493–503. 20041290 10.1007/s10571-009-9488-x PMC11498799

[ref49] Liu L. Daum C. Miguel Cruz A. Neubauer N. Perez H. Ríos Rincón A. (2022). Ageing, technology, and health: advancing the concepts of autonomy and independence. Healthc. Manage. Forum 35, 296–300. doi: 10.1177/08404704221110734 35924794 PMC9425715

[ref50] Logie R. H. Cocchini G. Delia Sala S. Baddeley A. D. (2004). Is there a specific executive capacity for dual task coordination? Evidence from Alzheimer’s disease. Neuropsychology 18, 504–513. doi: 10.1037/0894-4105.18.3.504 15291728

[ref51] Ly M. Karim H. T. Becker J. T. Lopez O. L. Anderson S. J. Aizenstein H. J.. (2021). Late-life depression and increased risk of dementia: a longitudinal cohort study. Transl. Psychiatry 11:147. doi: 10.1038/s41398-021-01269-y 33654078 PMC7925518

[ref53] Mandolesi L. Polverino A. Montuori S. Foti F. Ferraioli G. Sorrentino P.. (2018). Effects of physical exercise on cognitive functioning and wellbeing: biological and psychological benefits. Front. Psychol. 9:509. doi: 10.3389/fpsyg.2018.00509 29755380 PMC5934999

[ref54] Matysiak O. Kroemeke A. Brzezicka A. (2019). Working memory capacity as a predictor of cognitive training efficacy in the elderly population. Front. Aging Neurosci. 11:126. doi: 10.3389/fnagi.2019.00126 31214015 PMC6554703

[ref55] McDermott M. Liu K. Ferrucci L. Criqui M. H. Greenland P. Guralnik J. M.. (2006). Physical performance in peripheral arterial disease: A slower rate of decline in patients who walk more. Ann. Intern. Med. 144, 10–20. doi: 10.7326/0003-4819-144-1-200601030-00005 16389250

[ref56] Mertler C. A. Vannatta R. A. (2005). Advanced and multivariate statistical methods: practical application and interpretation (3.Basm). CA: Pyrczak Publishing.

[ref57] Moreno-Agostino D. Daskalopoulou C. Wu Y.-T. Koukounari A. Haro J. M. Tyrovolas S.. (2020). The impact of physical activity on healthy ageing trajectories: evidence from eight cohort studies. Int. J. Behav. Nutr. Phys. Act. 17:92. doi: 10.1186/s12966-020-00995-8 32677960 PMC7364650

[ref58] Nimrod G. Ben-Shem I. (2015). Successful aging as a lifelong process. Educ. Gerontol. 41:150616113530003. doi: 10.1080/03601277.2015.1050904

[ref59] Northey J. M. Cherubin M. Pumpa K. L. Smee D. J. Rattray B. (2018). Exercise interventions for cognitive function in adults older than 50: a systematic review with meta-analysis. Br. J. Sports Med. 52, 154–160. doi: 10.1136/bjsports-2016-096587 28438770

[ref60] Nuzum H. Stickel A. Corona M. Zeller M. Melrose R. J. Wilkins S. S. (2020). Potential benefits of physical activity in MCI and dementia. Behav. Neurol. 12:7807856. doi: 10.1155/2020/7807856 PMC7037481 32104516

[ref61] Palmiero M. (2015). The effects of age on divergent thinking and creative objects production: a cross-sectional study. High Abil. Stud. 26, 93–104. doi: 10.1080/13598139.2015.1029117

[ref62] Palmiero M. Nori R. Piccardi L. (2017). Verbal and visual divergent thinking in aging. Exp. Brain Res. 235, 1021–1029. doi: 10.1007/s00221-016-4857-4 28032140

[ref63] Panza G. A. Taylor B. A. MacDonald H. V. Johnson B. T. Zaleski A. L. Livingston J.. (2018). Can exercise improve cognitive symptoms of Alzheimer's disease? J. Am. Geriatr. Soc. 66, 487–495. doi: 10.1111/jgs.15241 29363108

[ref64] Pecchinenda A. Yankouskaya A. Boccia M. Piccardi L. Guariglia C. Giannini A. M. (2023). Exploring the relationship between perceived loneliness and subjective cognitive decline in older individuals. Aging Ment. Health, 4, 1–10. doi: 10.1080/13607863.2023.2242291 37540497

[ref65] Persson S. Saha S. Gerdtham U.-G. Toresson H. Trépel D. Jarl J. (2022). Healthcare costs of dementia diseases before, during and after diagnosis: longitudinal analysis of 17 years of Swedish register data. Alzheimers Dement. 18, 2560–2569. doi: 10.1002/alz.12619 35189039 PMC10078636

[ref66] Piccardi L. Nori R. Palermo L. Guariglia C. (2015). Age effect in generating mental images of building but not common objects. Neurosci. Lett. 602, 79–83. doi: 10.1016/j.neulet.2015.06.058 26149230

[ref67] Rajtar-Zembaty A. Rajtar-Zembaty J. Olszewska K. Epa R. Chrobak A. A. Starowicz-Filip A.. (2022). Comparison of cognitive functioning of elders with late-life depression and patients with and without a history of depressive episodes: a cross-sectional study. Psychol. Health Med. 27, 1227–1233. doi: 10.1080/13548506.2020.1859563 33351670

[ref68] Reitlo L. S. Sandbakk S. B. Viken H. Aspvik N. P. Ingerbrigtsen J. E. Tan X.. (2018). Exercise patterns in older adults instructed to follow moderate- or high-intensity exercise protocol-the generation 100 study. BMC Geriatr. 18:208. doi: 10.1186/s12877-018-0900-6 30200893 PMC6131829

[ref69] Rowe J. W. Kahn R. L. (1997). Successful aging. Gerontologist 37, 433–440. doi: 10.1093/geront/37.4.433 9279031

[ref72] Schmidt A. F. Finan C. (2018). Linear regression and the normality assumption. J. Clin. Epidemiol. 98, 146–151. doi: 10.1016/j.jclinepi.2017.12.006 29258908

[ref73] Serra L. Raimondi S. di Domenico C. Maffei S. Lardone A. Liparoti M.. (2021). The beneficial effects of physical exercise on visuospatial working memory in preadolescent children. AIMS Neurosci. 8, 496–509. doi: 10.3934/Neuroscience.2021026 34877401 PMC8611191

[ref74] Sheather S. A. (2009). Modern approach to regression with R; Springer New York, NY, USA.

[ref75] Singh B. Olds T. Curtis R. Dumuid D. Virgara R. Watson A.. (2023). Effectiveness of physical activity interventions for improving depression, anxiety, and distress: an overview of systematic reviews. Br. J. Sports Med. 57, 1–10. doi: 10.1136/bjsports-2022-106195 36796860 PMC10579187

[ref76] Sofi F. Valecchi D. Bacci D. Abbate R. Gensini G. F. Casini A.. (2011). Physical activity and risk of cognitive decline: a meta-analysis of prospective studies. J. Intern. Med. 269, 107–117. doi: 10.1111/j.1365-2796.2010.02281.x 20831630

[ref77] Song D. Yu D. S. F. Li P. W. C. Lei Y. (2018). The effectiveness of physical exercise on cognitive and psychological outcomes in individuals with mild cognitive impairment: A systematic review and meta-analysis. Int. J. Nurs. Stud. 79, 155–164. doi: 10.1016/j.ijnurstu.2018.01.002 29334638

[ref78] Strømnes D. G. Lars T. Kjetil S. Lødøen G. T. Magne B. T. (2013). Neuropsychological functioning in late-life depression. Front. Psychol. 4:2013. doi: 10.3389/fpsyg.2013.00381.Print PMC3694218 23818887

[ref003] Szychowska A. Drygas W. (2022). Physical activity as a determinant of successful aging: a narrative review article. Aging. Clin. Exp. Res. 34, 1209–1214. doi: 10.1007/s40520-021-02037-0 34873677 PMC9151514

[ref79] Troisi Lopez E. Liparoti M. Passarello N. Lucidi F. Mandolesi L. (2023). Multimodal physical exercise affects Visuo-spatial working memory: preliminary evidence from a descriptive study on tai-chi practitioners and runners. Brain Sci. 13:1400. doi: 10.3390/brainsci13101400 37891768 PMC10605525

[ref80] Vallat-Azouvi C. Pradat-Diehl P. Azouvi P. (2012). The working memory questionnaire: A scale to assess everyday life problems related to deficits of working memory in brain injured patients. Neuopsychol. Rehab. 22, 634–649. doi: 10.1080/09602011.2012.681110 22537095

[ref81] Weuve J. Kang J. H. Manson J. E. Breteler M. M. Ware J. H. Grodstein F. (2004). Physical activity, including walking, and cognitive function in older women. JAMA 292, 1454–1461. doi: 10.1001/jama.292.12.1454 15383516

[ref82] Wimo A. Jönsson L. Bond J. Prince M. Winblad B. (2013). The worldwide economic impact of dementia 2010. Alzheimers Dement. 9, 1–11. doi: 10.1016/j.jalz.2012.11.006 23305821

[ref83] Winter B. Breitenstein C. Mooren F. C. Voelker K. Fobker M. Lechtermann A.. (2007). High impact running improves learning. Neurobiol. Learn. Mem. 87, 597–609. doi: 10.1016/j.nlm.2006.11.003 17185007

[ref84] World Health Organization. (2020). “WHO fact sheet: physical activity.” Geneva.

[ref86] Wu J.-J. Wang H.-X. Yao W. Yan Z. Pei J.-J. (2020). Late-life depression and the risk of dementia in 14 countries: a 10-year follow-up study from the survey of health, ageing and retirement in Europe. J. Affect. Disord. 274, 671–677. doi: 10.1016/j.jad.2020.05.059 32664001

